# An Experimental Study of Subliminal Self-Face Processing in Depersonalization–Derealization Disorder

**DOI:** 10.3390/brainsci12121598

**Published:** 2022-11-22

**Authors:** Shanshan Liu, Yuan Jia, Sisi Zheng, Sitong Feng, Hong Zhu, Rui Wang, Hongxiao Jia

**Affiliations:** 1 The National Clinical Research Center for Mental Disorders, Beijing Key Laboratory of Mental Disorders, Beijing Anding Hospital, Capital Medical University, Beijing 100088, China; 2 Advanced Innovation Center for Human Brain Protection, Capital Medical University, Beijing 100088, China

**Keywords:** depersonalization/derealization disorder, subliminal, self-face processing, self-face advantage, continuous flash suppression

## Abstract

The self-perception or self-experience of patients with depersonalization/derealization disorder (DPD) is altered, leading to a profound disruption in self-awareness. The main aim of the study is to explore the characteristics of subliminal self-face processing in DPD patients. To our knowledge, this is the first experimental study that has measured and evaluated subliminal self-processing in DPD. To better understand this, we examined the ability of patients with DPD and healthy controls (HC) to identify pictures of faces using an experimental paradigm of breaking continuous flash suppression. There were 23 DPD outpatients from Beijing Anding Hospital, Capital Medical University and 23 matched HC who participated in this experiment. The time needed for a face to break into awareness was taken as the measure of participants’ subliminal processing of that face. The results indicated that there were significant differences between the DPD patients and HC in subliminal reaction times to different faces. Under experimental conditions, the average reaction response of self-face recognition in the HC group was significantly faster than for a famous face. However, this difference was not observed in DPD patients, which means that DPD patients did not show the processing advantage of their own faces as did the HC. The results suggest a deficit in subliminal self-face processing in DPD.

## 1. Introduction

Depersonalization disorder and depersonalization/derealization disorder (DPD) are now considered synonymous [1]. Depersonalization refers to a state in which the sense of self and the quality of subjective first-person experiences are oddly altered [2]. One of its core clinical features is a sense of detachment—a feeling that the self is detached from one’s own body. Examples include the feeling of being detached from one part of the body or the whole body, feeling estranged from one’s own image when looking in the mirror, being unable to control one’s movements, feeling disconnected from one’s surroundings, being separated from the world like a veil, or the feeling that the individual is an outside observer [3]. It has been suggested that this sense of disengagement or unreality is due to a misintegration of self-perception and self-awareness or stems from a change in the patient’s self-perception or self-experience or the disturbance of self–other/self–world boundaries [4,5].

The “self” is unique and different from others. The distinctiveness of the self is reflected in both the perception of and behavioral responses to the self. The ability to identify oneself and distinguish between ‘self’ and ‘not-self’ is critical to many higher-order cognitive abilities, such as self-awareness and theory of mind [6]. Healthy individuals exhibit a “self-advantage” in recognizing self-related information, which tends to be processed faster and more accurately than other types of stimuli. This “self-advantage” has been observed in various studies on the self, such as a person’s name, expression, and face [7,8,9]. The ability to recognize one’s own face has been suggested to be an indicator of self-awareness relative to other self-related stimuli [10]. Investigating individuals’ self-face recognition may help us better in understanding the processing of self-related information, and the self-face paradigm has been used to examine the neural basis of the self [11,12].

DPD, a chronic disorder characterized by a profound disruption of self-consciousness, appears to be more common than previously thought [13]. However, current research on DPD is not as extensive as it is for other conditions such as schizophrenia and depression [14]. It is reported that the prevalence rate of DPD is around 1% in the general population [15]. Ketay et al. [16] proposed the positive relationship between depersonalization severity and brain activation in the medial prefrontal cortex (MedPFC) which also points to a deficit in implicit self-processing, and there might be a conflict between conscious and unconscious processing involving the self in DPD. Farmer et al. [17] used the visual remapping of the touch paradigm and reported that people with a higher rate of depersonalization experiences do not show the typical pattern of self-face bias when integrating visual–tactile information. Due to differences in control stimuli (familiar, unfamiliar, and feature judgments) and experimental tasks (e.g., photo/stimuli presentation, and implicit vs. explicit tasks), interpreting and integrating these activation mechanisms involving the self has proven to be difficult [18]. In addition, since most previous studies have been limited to supraliminal consciousness, it is necessary to examine the self-face processing in DPD patients especially when the face stimuli are at the subliminal perceptual level, to address the question: ‘Are there any differences between DPD patients and the healthy controls (HC) in self-face processing?’

At present, methods exist to make the stimuli at the subliminal level including the visual masking paradigm, reducing the intensity of stimuli, or reducing the perception of subjects. In order to induce stable behavioral effects, this study adopted a highly controllable experimental paradigm, continuous flash suppression (CFS), which has been increasingly used in studies of unconscious processing [19,20]. CFS is a new experimental stimulus presentation technique based on the improvement of binocular competition [21,22], in which a set of dynamic noise images are presented to the subject's dominant eye, while a face image with gradually increasing contrast is presented to his/her non-dominant eye. This combination creates interocular suppression between the eyes and leads to the conscious perception of noise images, while the face image is initially invisible. With the extension of time and increased contrast in the face image, it eventually breaks the suppression and enters consciousness and is perceived and recognized. Therefore, the time needed for a face to break into awareness is defined as the measure of participants’ subliminal processing of that face. Assuming that subjects have a subliminal processing advantage for self-face, the time required for self-face to break through the suppression between eyes and enter consciousness is significantly faster than that for other-faces. In contrast, if the subliminal processing of self-face lacks an advantage, the self-face stimuli would not break this suppression more quickly, and a significant difference in the response to the self-face and familiar-face stimuli would not be observed. In view of the above assumptions, the research purpose is to explore the characteristics of subliminal self-face processing and whether there are deficits in subliminal self-face processing in DPD patients.

## 2. Materials and Methods

### 2.1. Participants and Settings

Twenty-three patients with DPD were included in this study. They were recruited from the outpatient unit of Beijing Anding Hospital, Capital Medical University, between June 2017 and October 2018. They were diagnosed as having depersonalization/derealization disorder by at least two attending physicians according to the DSM-V. All patients with DPD who participated in this experiment did not receive any pharmacological treatment (antidepressants, benzodiazepines, etc.). Twenty-three HC were recruited from Beijing Anding Hospital, Capital Medical University, universities or colleges, and local communities.

DPD patients and the HC were required to meet the following inclusion criteria: (1) age 16–45 years old; (2) education level of junior high school or above; (3) normal binocular vision or corrected vision; (4) able to recognize the Chinese president from the photos provided before participating in the experiment; (5) no obvious and easily recognizable marks on the face, such as tattoos, scars and moles, etc.; and (6) able to understand and complete the requirements of this experiment. In addition, patients with DPD were required to have a score of ≥70 on the Cambridge Depersonalization Scale (CDS) [23]. Additional inclusion criteria for the HC group were: (1) did not meet any of the diagnostic criteria for a DSM-V mental disorder; (2) had no diagnosis of depression, anxiety disorder or other mental disorders by screening through Mini-International Neuropsychiatric Interview (MINI); and (3) had no positive family history of mental illness.

The patients were excluded from the study if they had: (1) a history of psychoactive substance abuse, chronic neurological disease, mental retardation, or other serious physical illnesses; (2) had any other comorbid psychiatric diagnosis (e.g., depression, anxiety disorders or obsessive–compulsive disorder) through the MINI assessment; (3) had received electroconvulsive therapy within the past 6 months; (4) the patient was currently at risk of being excited, impulsive, or engaging in self-injury according to the judgment of investigator; and (5) the informed consent form had not been signed.

### 2.2. Ethics Approval

This study was approved by the Ethics Committee of Beijing Anding Hospital, Capital Medical University, Ethical Approval Code: 2020-ky-17. Written, informed consent was obtained from the participant if they were 18 years or older, or from their parent (with the participant’s written assent) if the participant was under 18 years of age.

### 2.3. Clinical Assessment

According to the research characteristics and requirements, a general information questionnaire was developed to collect demographic data and clinical characteristics, including gender, age, years of education, and duration of DPD. The research physician who completed the training of the research protocol used a set of standardized instructions to guide participants in completing the CDS and the Rosenberg Self-Esteem Scale (SES). The Cambridge Depersonalization Scale is a reliable and effective tool for screening for DPD. The patients with DPD fulfill the Chinese versions of the CDS prior to the face recognition. The Chinese version of the CDS was confirmed to have good reliability and validity in community samples in China [24]. The scale focuses on the frequency and duration of select abnormal experiences, with a total score of 0–290. The higher the score, the higher the detection rate of DPD. The Rosenberg Self-Esteem Scale is a tool for measuring self-esteem, which is used to assess overall feelings of self-worth and self-acceptance. The scale consists of 10 items, of which 5 items are positively scored and 5 items are scored in reverse. The total score is between 10–40, with higher scores indicating higher self-esteem. The Chinese version of SES translated by Cheng et al. and used in this study has also been used in previous studies [25,26]. The questionnaires were completed independently by participants on site.

### 2.4. Experimental Stimuli

Three kinds of face stimuli were used in this study, the subject’s own face, a familiar face and a stranger’s face. Under the same lighting conditions, a neutral frontal photo of each participant was taken by a research assistant using a digital camera as the self-face stimuli before the experiment. To reduce the potentially confounding influence of differences between participants in their familiarity with famous faces, we selected the picture of the Chinese president, with which we could be reasonably certain that all participants were familiar, as the famous face stimulus. The faces of people who the participants had never seen were selected to be the unfamiliar face stimuli. All pictures were standardized to balance the distance between the two pupils and the vertical midline of the face. Then, each modified image was resized to 256 × 256 pixels (5.51 × 5.51 viewing angle) and matched for brightness (luminance = 3.7 cd/mm^2^) and contrast (root mean square-contrast = 0.51). Final low-contrast blue face images were produced by removing the red and green color channels of the images, and they were presented against a gray background (128, 128, 128 RGB). The “mosaic” image was a scrambled unrecognizable face, consisting of 169 pieces (20 × 20 pixels).

**Figure 1:**
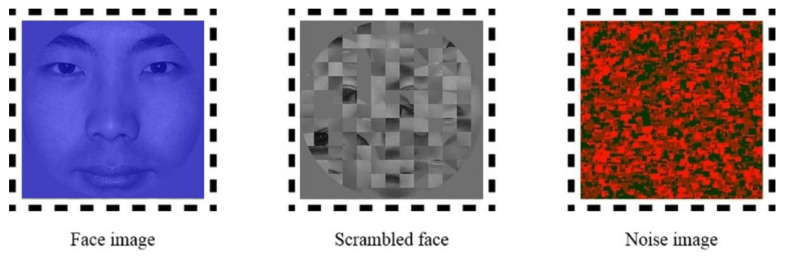
Experimental stimuli images.

**Figure 2:**
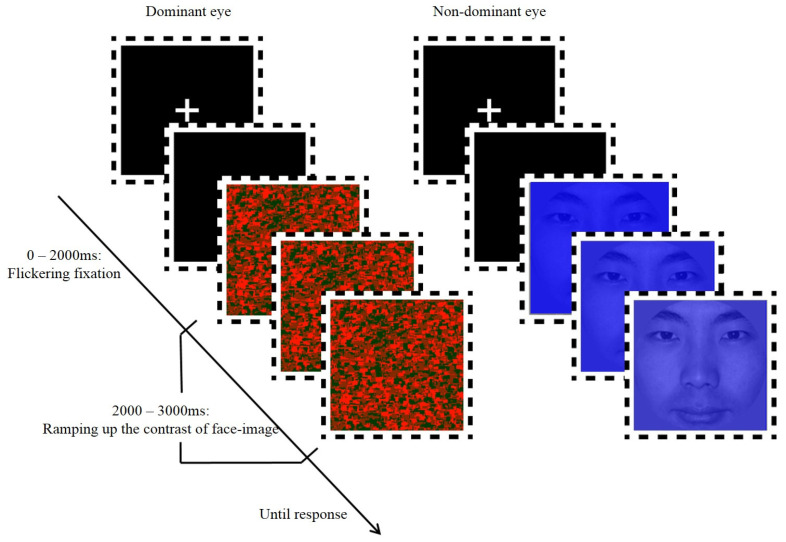
Illustration of a trial in the experimental condition.

The dynamic noise images matching the face stimuli were created by OpenGL 2.0 (Silicon Graphics International Corp., Fremont, CA, USA). Each set of noise images consisted of randomly positioned squares of various sizes covering the entire image area, and each square was composed of a mixture of red and green squares with different proportions. In each successive noise image, 10% of the area of the previous picture was replaced by new squares at random locations, resulting in a subsequent noise image. To produce more stable interocular suppression, each trial noise image was presented continuously at 100 Hz instead of 10 Hz as commonly used [27,28]. The face images and dynamic noise images were presented in a frame in the center of a computer screen (Figure 1). The dynamic noise images were always presented with full contrast, while the contrast of the face images was gradually increased from 0% to 100% (the alpha channel value was from 0 to 1) in each trial. The face image presented in Figure 1 and Figure 2 was a volunteer who has signed informed consent for publication.

### 2.5. Experimental Procedure

All participants were tested in a soundproof laboratory and were instructed to complete the experiment using standardized instructions. The experiment officially started when the participant fully understood the experimental requirements and had completed practice exercises until he/she was confident in completing the task. During the experiment, the head of the subject was fixed with a chin rest so that his/her eyes were 60 cm away from the computer screen. In addition, this experiment consisted of two blocks, one of which was the experimental condition and was completed first by subjects, followed by the control condition. The trials within each block were random.

In the experimental block, participants wore a pair of red–blue anaglyph glasses so that the noise images were only visible to their dominant eye through the red filter lens, and simultaneously, the face image was only visible to their other eye through the blue filter lens. The resulting interocular suppression consequently allowed participants to see continuous presentations of the noise images but were not aware of the existence of the face image until it broke into awareness. Participants did not wear red–blue anaglyph glasses in the control condition and the dynamic noise images and the face images were presented between the two eyes at the same time so that there was no binocular suppression.

Each experimental block consisted of 55 trials, including 15 self-face pictures, 15 identical famous pictures, 15 stranger pictures, and 10 scrambled faces. The scrambled pictures were presented to the non-dominant eye, and if the subject had a keystroke response within 10 s, it was marked as an error; if the false alarm was reached three times, the trial was restarted. The scrambled pictures and face pictures were randomly presented in each trial.

Before the start of each trial, a white fixed point “+” was presented in the center of the screen that flashed continuously for 2000 ms to attract the subjects’ attention. In the experimental condition, the subjects’ dominant eye was always presented with a 100% contrast dynamic noise image, while his/her non-dominant eyes were presented with face pictures and scrambled pictures. The contrast of the latter two kinds of pictures was gradually changed from 0 to 100% within 1000 ms and remained at 100% for the next 9000 ms until the end of the trial. In the control condition, the subjects’ eyes were presented with dynamic noise images, face pictures and scrambled pictures that varied from 0 to 100% and 100% contrast was maintained for 4000 ms because the binocular presentation of stimuli may make the task easier. Participants were asked to press the space bar as quickly as possible once they recognized the face image or any part of it. The face and noise images remained on the screen until either a response was recorded or 10 s had elapsed (See Figure 2).

### 2.6. Statistical Analysis

After the experiment, a result was considered invalid if the participant had responded to a scrambled picture three or more times, and was excluded from the analysis. Similarly, trials with no responses or with extreme reaction times (more than three standard deviations from the mean) were not included in the analysis.

Our study adopted a 2 (Group: DPD vs. HC) × 2 (Condition: experimental condition vs. control condition) × 3 (Task: self vs. famous vs. stranger) mixed factorial design. The independent variables were task and group; the dependent variable was the time required for a face to break through the interocular inhibition and enter consciousness. Data were entered into IBM SPSS Statistics for Windows, version 25.0 (IBM Corp., Armonk, NY, USA) and then analyzed. The *t*-test was used to compare the two groups of quantitative data that conformed to a normal distribution and the χ^2^ test was used to compare rates of qualitative data between groups. A comparison of the average reaction times of the two groups in recognizing the three kinds of face pictures under different blocks was performed using a repeated measures analysis of variance (ANOVA). The pairwise comparisons were performed using the least significance difference (LSD) test. Finally, we conducted Pearson correlation analysis to assess associations between the reaction times of patients with DPD to the self-face stimuli with age, duration of DPD, CDS scores and SES scores, respectively. The level of statistical significance was set as *p* < 0.05.

## 3. Results

### 3.1. Demographic Information and Clinical Data

Twenty-three patients with DPD and 23 HC were included in the final statistical analysis. Table 1 describes some demographic information and clinical characteristics. As shown, there were no significant differences in gender, age or education between the two groups (*p* > 0.05). However, there was a significant difference in SES scores (*p* = 0.001).

### 3.2. The Comparison of Reaction Times between DPD and HC in Recognizing Different Faces

The results of the repeated measurement ANOVA showed that the main effect of “Group” (*F* (1, 44) = 4.498, *p* = 0.040), “Task (face type)” (*F* (2, 88) = 22.259, *p* = 0.000), and “Condition” were all significant (*F* (1, 44) = 8.545, *p* = 0.005). The interaction between “Task” and “Condition” was also significant (*F* (2, 88) = 3.444, *p* = 0.036). In addition, the interaction between the two groups of participants in recognizing different faces under different conditions was significant (*F* (2, 88) = 4.771, *p* = 0.011). See Table 2.

The results of the analysis of the simple effects within the test groups showed that the main effect of “Task” was significant (*F* (2, 44) = 13.465, *p* = 0.000) for the HC group, and the interaction between “Task” and “Condition” was significant (*F* (2, 44) = 3.913, *p* = 0.027). Detection times for self-face were significantly faster than that for famous faces (*p* = 0.013) and stranger’s faces (*p* = 0.000) for the HC in the experimental condition. In comparison, detection times for self-face were also significantly faster than for stranger’s faces (*p* = 0.003), in the control condition but no significant difference was observed between self-face and famous faces (*p* > 0.05).

**Figure 3:**
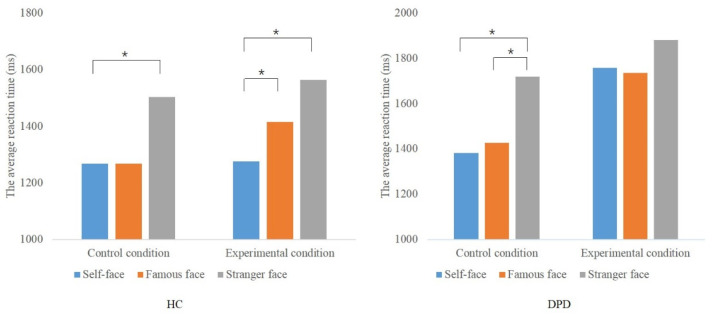
The reaction times to the self-face, famous faces and strangers’ faces for the HC and DPD, for the control and experimental conditions; HC: Healthy controls; DPD: Depersonalization/derealization disorder; * *p* < 0.05.

For the DPD group, the main effect of “Task” (*F* (2, 44) = 9.548, *p* = 0.000) and “Condition” (*F* (1, 22) = 14.229, *p* = 0.001) were significant, and the interaction between “Task” and “Condition” was also significant (*F* (2, 44) = 4.189, *p* = 0.022). For the DPD patients, the average reaction times to recognize self-face, famous faces and stranger’s faces were not different (*p* > 0.05) in the experimental condition. However, it is worth noting that although the detection times for self-face and famous faces were not statistically different, the recognition speed of self-face was slightly slower than for famous faces for DPD patients, which was contrary to the HC. In the control condition, although the patient’s recognition time for self-face (*p* = 0.000) and famous faces (*p* = 0.001) were significantly faster than for stranger’s faces, there was no significant difference in the detection speed of self-face and famous faces (*p* > 0.05). See Figure 3.

### 3.3. Correlation Analysis

**Figure 4:**
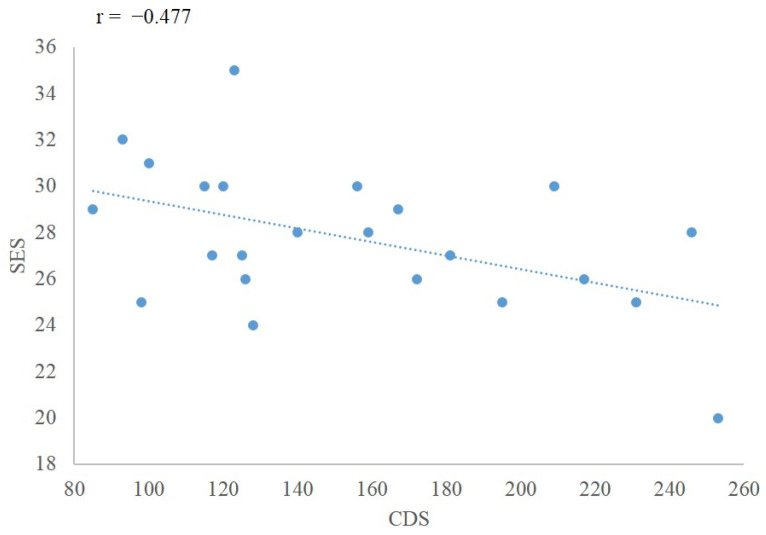
The correlation between CDS and SES scores in DPD; CDS: Cambridge Depersonalization Scale; SES: Rosenberg Self-Esteem Scale; DPD: Depersonalization/derealization disorder.

The statistical analysis showed a moderate negative correlation between CDS and SES scores (*p* = 0.021). See Figure 4.

The results of the correlational analysis showed that there were no significant correlations between subliminal self-face processing and age, duration of DPD, CDS scores, or SES scores (*p* > 0.05). See Table 3.

## 4. Discussion

There is sufficient evidence to demonstrate that healthy individuals have an advantage in processing images of their own faces, most of which are processed at the level of supraliminal consciousness [29,30]. To date, while there are some applications of subliminal self-face processing or visual processing in healthy people and patients with schizophrenia, there is a lack of research data on DPD. This study is the first to explore the characteristics of subliminal self-face processing in patients with DPD.

This study found that the self-esteem of DPD patients was lower than that of the HC. At the same time, it showed that the CDS score was negatively correlated with the SES score, indicating that the higher the score of CDS, the lower the self-esteem level. Self-esteem usually refers to the feeling of self-worth and the evaluation and acceptance of self-value and self-importance [31], and there is relatively little research on the self-esteem of patients with DPD. Consistent with this study, Michal et al. [32] found that patients with DPD often feel helpless, vulnerable, worthless and isolated from society, which may lead to low self-esteem. Hedrick et al. [33] found that implicit self-esteem was impaired in DPD and that patients with DPD had a stronger sense of self-separation compared with the HC. The decreased emotional intensity and self-esteem experienced by patients with DPD depended on their internal self-perception. When they evaluated themselves, they did not think it was their own evaluation but that of a bystander, leading to abnormal self-cognition. They lack the ability to accurately perceive themselves, which promotes their negative evaluation of themselves. The poorer self-esteem of patients with DPD may be related to changes in the quality of their consciousness (interruption or destruction of self-consciousness, abnormal subjective experience). 

In the control condition, i.e., without interocular inhibition, we did not find differences in detection times for the self-face and famous faces in healthy subjects. However, it took significantly less time for self-face to break the interocular inhibition and enter the HC’s consciousness level than for famous faces and strangers’ faces in the experimental condition. This finding was consistent with previous research results [22,34,35] and suggests that the HC had an advantage in subliminal self-face processing. In addition, there was no significant difference between their reaction times when recognizing their own faces compared with famous faces and strangers’ faces. It is worth noting that not only was there no such difference, but the recognition speed of patients with DPD in response to self-face was also slightly slower than to famous faces, which was contrary to the HC. According to current results, we could infer that DPD patients lack the self-processing advantage at the subliminal consciousness level [16,17].

The self-face represents a unique feature, and processing self-face shows an advantage over other self-related stimuli [9,36]. Self-face may be automatically detected in the early stage of visual processing because it requires few cognitive resources and involves little cortical processing [37]. The damage to self-concept or self-experience of patients with DPD is reflected in abnormal subliminal self-face processing, which may be the basis for their feeling of self-separation or unfamiliarity with themselves. The self-deficit leads to their inability to give priority to self-related processing, and an inability to respond quickly to self-face images in this experiment. This self-processing disorder seems to be consistent with clinical symptoms, such as the unreality about themselves or the world around them, the strangeness of their own memory, and abnormal subjective self-experiences. This deficit tended to be a characteristic marker and was not related to the age, duration of DPD, CDS score, or SES score of the patients. The difficulty that patients with DPD have in integrating their visceral and bodily perceptions into a sense of themselves may be due to specific impairments in interoceptive self-awareness and body perception [38,39].

There may be some neural basis for the deficits in subliminal self-processing in DPD. Brain imaging studies in DPD have revealed widespread alterations in metabolic activity in the sensory association cortex, as well as prefrontal hyperactivation and limbic inhibition in response to aversive stimuli [40]. A recent fMRI study [41] found that subjects with DPD had stronger frontal lobe activity when responding to self-face images compared to strangers’ faces. These differences might reflect defects in implicit self-processing. Consistent with this result, Ketay et al. [16] thought that participants with DPD exhibited greater responses to self compared to stranger processing within the right anterior cingulate cortex, bilateral medial prefrontal cortex, and left middle frontal gyrus, and depersonalization severity was significantly associated with activation of the latter two regions. Therefore, the symptoms of loss of self-perception and separation are closely related to the self-processing disorder in DPD. Sierra and Berrios proposed a new model that the state of increased alertness observed in depersonalization results from an activation of prefrontal attentional systems (right dorsolateral prefrontal cortex) and reciprocal inhibition of the anterior cingulate, leading to experiences of “mind emptiness” and “indifference to pain” often seen in DPD [42]. Two other structural MRI studies have been conducted with large samples of patients with DPD and the HC. The results indicated that changes in gray matter were the basis of DPD symptomatology. In one case [43], the right middle temporal gyrus of DPD was found to have a smaller cortical thickness; in another case [44], the volume of gray matter in the right caudate nucleus, right thalamus and right thalamus decreased, while the volume of left dorsomedial prefrontal cortex and right somatosensory region increased. Other studies have pointed out that activity of the right posterior insula was consistently implicated in self-attribution and self-processing, and the representation of an egocentric reference frame [45,46,47]. A study that focused specifically on patients with somatoparaphrenic symptoms revealed that the right posterior insula is indeed the critical structure involved in the phenomena of “disturbed sensation of limb ownership” [48]. However, the neuroimaging evidence related to self-deficits in patients with DPD requires further evaluation.

In the current design, we included a control condition that presented the same face stimuli as in the experimental condition, except that the control condition did not involve interocular suppression. Since there were no significant reaction time differences when responding to the self-face versus the famous faces in the control condition (for both DPD patients and the HC), we can exclude the possibility that findings in the experimental condition were simply due to the physical characteristics of the face stimuli [22].

## 5. Limitations

There were some limitations of this study. First, the sample size was relatively small, because it is difficult to recruit large samples of patients with this disorder (as it is rare and somewhat challenging to diagnose). However, a notable result was obtained, that is, subliminal self-face processing is impaired in DPD. The expansion of the sample size and classifying the symptoms to obtain more accurate behavioral results in future studies will be considered. Secondly, the type and dosage of medication in the follow-up analyses were not a control, and hence we cannot be certain whether these factors impacted the results. Finally, due to limitations of human resources and other factors, we were only able to conduct a preliminary behavioral analysis, which may be further studied in combination with functional magnetic resonance imaging, repetitive transcranial magnetic stimulation, or other methods in the future.

## 6. Conclusions

This study showed that the self-esteem of DPD patients was impaired compared with the HC, and patients with DPD exhibited deficits in subliminal self-face processing. The latter might prefer to be a characteristic marker and was not correlated with age, duration of DPD, frequency of depersonalization experiences, or level of self-esteem. Our study contributes experimental data to better understand subliminal self-processing in DPD. In the future, the conduct of in-depth research on self-processing in combination with neuroimaging or electrophysiology is required to provide a greater understanding of the neural substrates associated with these deficits in this patient population and to suggest effective clinical interventions.

## Figures and Tables

**Table 1:** Demographic information and clinical data in DPD patients and the HC.

Variables	DPD (*n* = 23)	HC (*n* = 23)	t/χ^2^	*p*
Gender (Male/Female)	14/9	8/15	3.136	0.139
Age (years)	25.87 ± 7.24	25.52 ± 2.50	−0.218	0.829
Education (years)	14.39 ± 2.21	15.30 ± 1.55	1.622	0.113
Duration of DPD (months)	7.43 ± 7.84	N/A	N/A	N/A
CDS	154.61 ± 50.52	N/A	N/A	N/A
SES	27.73 ± 3.11	31.43 ± 3.65	3.695	0.001 *

DPD: Depersonalization/derealization disorder; HC: Healthy controls; CDS: Cambridge Depersonalization Scale; SES: Rosenberg Self-Esteem Scale; Data are presented as means ± SD; The higher the SES score, the higher the level of self-esteem; * *p* < 0.05.

**Table 2:** The reaction times of patients with DPD and the HC when recognizing different faces.

Task (Face Stimuli)	Block	DPD	HC
Self-face	Experimental condition	1.76 ± 0.66	1.28 ± 0.32
	Control condition	1.38 ± 0.35	1.27 ± 0.50
Famous face	Experimental condition	1.74 ± 0.67	1.41 ± 0.33
	Control condition	1.43 ± 0.53	1.27 ± 0.40
Stranger's face	Experimental condition	1.88 ± 0.54	1.56 ± 0.47
	Control condition	1.72 ± 0.52	1.50 ± 0.64

DPD: Depersonalization/derealization disorder; HC: Healthy controls.

**Table 3:** Results of the correlational analysis between subliminal self-face processing and other variables in patients with DPD.

	Self-Face			
	Control Condition		Experimental Condition	
	r	*p*	r	*p*
Age	0.07	0.77	0.11	0.63
Duration of DPD	−0.16	0.46	−0.04	0.85
CDS score	0.08	0.71	0.04	0.87
SES score	0.14	0.54	0.23	0.28

DPD: Depersonalization/derealization disorder; CDS: Cambridge Depersonalization Scale; SES: Rosenberg Self-Esteem Scale.

## Data Availability

Not applicable.
